# Carvacrol Attenuates Diabetic Cardiomyopathy by Modulating the PI3K/AKT/GLUT4 Pathway in Diabetic Mice

**DOI:** 10.3389/fphar.2019.00998

**Published:** 2019-09-12

**Authors:** Ning Hou, Yunpei Mai, Xiaoxia Qiu, Wenchang Yuan, Yilang Li, Chengfeng Luo, Yun Liu, Guiping Zhang, Ganjiang Zhao, Jian-dong Luo

**Affiliations:** ^1^Key Laboratory of Molecular Target & Clinical Pharmacology, School of Pharmaceutical Sciences and the Fifth Affiliated Hospital, Guangzhou Medical University, Guangzhou, China; ^2^Guangzhou Institute of Cardiovascular Disease, Guangzhou Key Laboratory of Cardiovascular Disease, the Second Affiliated Hospital of Guangzhou Medical University, Guangzhou, China; ^3^Department of Medical Technology, Forevergen Biosciences Center, Guangzhou, China; ^4^Department of Clinical Laboratory, the Fifth Affiliated Hospital of Guangzhou Medical University, Guangzhou, China

**Keywords:** diabetes mellitus, cardiac remodelling, carvacrol, cardiac function, PI3K/AKT pathway

## Abstract

**Background:** Diabetic cardiomyopathy (DCM), a common complication of diabetes mellitus, eventually leads to heart failure. Carvacrol is a food additive with diverse bioactivities. We aimed to study the protective effects and mechanisms of carvacrol in DCM.

**Methods:** We used a streptozotocin-induced and *db/db* mouse model of types 1 and 2 diabetes mellitus (T1DM and T2DM), respectively. Both study groups received daily intraperitoneal injections of carvacrol for 6 weeks. Cardiac remodeling was evaluated by histological analysis. We determined gene expression of cardiac remodeling markers (*Nppa* and *Myh7*) by quantitative real-time PCR and cardiac function by echocardiography. Changes of PI3K/AKT signaling were determined with Western blotting. GLUT4 translocation was evaluated by Western blotting and immunofluorescence staining.

**Results:** Compared with control mice, both T1DM and T2DM mice showed cardiac remodeling and left ventricular dysfunction. Carvacrol significantly reduced blood glucose levels and suppressed cardiac remodeling in mice with T1DM and T2DM. At the end of the treatment period, both T1DM and T2DM mice showed lesser cardiac hypertrophy, *Nppa* and *Myh7* mRNA expressions, and cardiac fibrosis, compared to mice administered only the vehicle. Moreover, carvacrol significantly restored PI3K/AKT signaling, which was impaired in mice with T1DM and T2DM. Carvacrol increased levels of phosphorylated PI3K, PDK1, AKT, and AS160 and inhibited PTEN phosphorylation in mice with T1DM and T2DM. Carvacrol treatment promoted GLUT4 membrane translocation in mice with T1DM and T2DM. Metformin was used as the positive drug control in T2DM mice, and carvacrol showed comparable effects to that of metformin on cardiac remodeling and modulation of signaling pathways.

**Conclusion:** Carvacrol protected against DCM in mice with T1DM and T2DM by restoring PI3K/AKT signaling-mediated GLUT4 membrane translocation and is a potential treatment of DCM.

## Introduction

Diabetic cardiomyopathy (DCM) is a cardiovascular disease (CVD) unique to patients with diabetes mellitus (DM). DCM is characterized by structural and functional cardiac alterations in the absence of traditional risk factors of heart failure, such as hypertension and valvular heart disease (Jia et al., 2018). Chronic DM leads to left ventricular hypertrophy, myocardial fibrosis, and diastolic dysfunction in early-stage DCM, as well as systolic dysfunction and subsequent heart failure in late-stage DCM. Despite the magnitude of consequences from DCM in patients with DM (Dandamudi et al., 2014), there is a lack of effective pharmacotherapy for DCM.

The etiopathogenesis of DCM has been attributed to the impairment of cardiac metabolism secondary to decreased glucose uptake and metabolism in both type 1 diabetes mellitus (T1DM) and type 2 diabetes mellitus (T2DM) (Jia et al., 2018). In T1DM and T2DM, respectively, insufficient insulin production or insulin resistance causes a significant reduction of glucose uptake. Moreover, the insulin dysfunction and hyperglycemia in DM induce oxidative stress inhibit coronary nitric oxide generation and increase production of advanced glycation end products (AGEs) (Jia et al., 2018). These harmful factors potentially lead to intracellular calcium overload, with resultant cardiac stiffness and diastolic dysfunction (Murtaza et al., 2019). The impaired utilization of glucose in cardiomyocytes is accompanied by downregulation of glucose transporters (GLUTs). GLUT type 4 (GLUT4) is the major cardiac isoform of GLUTs, which accounts for approximately 70% of all cardiac GLUTs (Mueckler and Thorens, 2013). The expression and membrane translocation of GLUT4 decreased in the cardiac muscle of both—animals with T1DM and T2DM (Camps et al., 1992; Desrois et al., 2004). GLUT4 is regulated by the insulin-stimulated phospatidylinositol 3-kinase (PI3K)/AKT signaling pathway, which is impaired in diabetes; this phenomenon is considered responsible for the pathogenesis of DCM (Huang et al., 2009). AKT modulates insulin-regulated glucose metabolism, which facilitates GLUT4 translocation from cellular stores to the plasma membrane (Kohn et al., 1996). The activation of PI3K/AKT signaling is beneficial for the treatment of DCM (Qi and Zhong, 2018).

**Figure 1:**
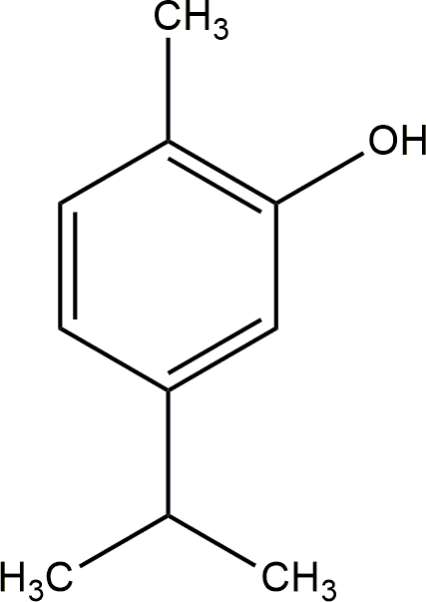
Chemical structure of carvacrol.

Natural product drug discovery has, over the past few years, become an important focus of studies on novel cardioprotective compounds that are potential drug candidates for DM and DM-associated cardiovascular complications. Carvacrol (**Figure 1**) is a Food and Drug Administration (FDA)–approved food additive. Carvacrol is an essential monoterpenic phenol that is isolated from various mints. Carvacrol modulates a variety of bioactivities, including antioxidation (Chenet et al., 2019), anti-inflammation (Chenet et al., 2019), TRPM7 ion channel inhibition (Parnas et al., 2009), as well as antimicrobial (Mbese and Aderibigbe, 2018) and antitumor activities (Mbese and Aderibigbe, 2018). After carvacrol administration in mice with high-fat-diet- or streptozotocin (STZ)–induced DM, serum total cholesterol and blood glucose levels decreased (Bayramoglu et al., 2014; Ezhumalai et al., 2014), diabetes-induced cognitive deficits improved (Deng et al., 2013), and oxidative stress and cell apoptosis in testicular tissue were ameliorated (Shoorei et al., 2019). However, the specific mechanism by which carvacrol reduces blood glucose in DM, and the effects of carvacrol on DCM remain unknown.

This study was conducted to investigate the effects of carvacrol on DCM and the PI3K/AKT/GLUT4 signaling pathway in both mice with T1DM or T2DM.

## Materials and Methods

### Reagents

Carvacrol, Metformin and STZ were purchased from Sigma Aldrich (St. Louis, MO, USA). The inhalational anesthetic, isoflurane, was purchased from RWD Life Science (China). The Mem-PER^™^ Plus Membrane Protein Extraction Kit, Pierce^™^ BCA Protein Assay Kit, SuperSignal^™^ West Pico Chemiluminescent Substrate, and TRIzol^®^ Reagent were purchased from Thermo Fisher Scientific (Rockford, IL, USA). The RIPA lysis and extraction buffer were procured from Cell Signaling Technology (Danvers, MA, USA). The protease and phosphatase inhibitors were purchased from BioTool (Houston, TX, USA). The First-Strand cDNA Synthesis Kit was purchased from Takara (Japan). All other chemical reagents were of analytical grade and obtained from Dingguo Biotechnology Company (China).

### Animals

Adult male C57BL/6J mice (6 to 7 weeks old) were purchased from the Guangdong Medical Laboratory Animal Center (Guangzhou, China). Adult male C57BLKS/J Iar-+ *lepr* *^db^*/ + *lepr* *^db^* (*db/db*) mice were purchased from the Nanjing Biomedical Research Institute of Nanjing University. All experimental animals were housed in individual cages and a controlled environment (12:12-h day/night cycle, 50–70% humidity, 24°C) with *ad libitum* food and water. We undertook efforts to minimize the stress to the animals. All animal handling and treatments were conducted strictly according to the EC Directive 86/609/EEC. The animal use and care protocols were reviewed and approved by the ethics committee of Guangzhou Medical University.

### Induction and Assessment of T1DM in Mice

The mice received intraperitoneal (i.p.) injections of STZ (45 mg/kg/day) for five consecutive days, as described previously (Qin et al., 2016). Age-matched male C57 mice were used as the control group and injected with an equivalent volume of citrate buffer (pH 4.4, concentration 0.1 mol/L). Seventy-two hours after the final injection, blood glucose was determined using a glucose analyzer (OneTouch Ultra Mini Blood Glucose Monitoring System, Johnson Co., China) through tail vein. Animals with blood glucose levels higher than 16.7 mmol/L were considered diabetic and included in the subsequent experiments.

### Experimental Schema for Mice With STZ-Induced T1DM

Four weeks after induction of DM, the T1DM mice were randomly divided into three groups: 1) the diabetic group (T1DM, n=8), which received i.p. injections of 0.1% dimethyl sulfoxide (DMSO); 2) the diabetic group treated with carvacrol 10 mg/kg/day (T1DM+CAR10, n=10); and 3) the diabetic group treated with carvacrol 20 mg/kg/day (T1DM+CAR20, n=10). Carvacrol was freshly reconstituted before use and administered *via* i.p. injections to the animals once a day consecutively for 6 weeks. The control mice received 20 mg/kg/day CAR (Con+CAR20, n=10) or equivalent volume of 0.1% DMSO (Con, n=8) *via* i.p. injections for 6 weeks. All mice were fed a normal diet and could freely access drinking water during the entire course of the experiment. The bodyweight and random blood glucose of mice in each group were measured every 2 weeks. Random blood glucose was measured using the OneTouch Ultra glucometer through tail vein. At the end of the experiment, all mice underwent an echocardiographic assessment; thereafter, the mice were anesthetized with i.p. ketamine (80 mg/kg)/xylazine (8 mg/kg) and then sacrificed. Cardiac tissues were collected and stored at −80°C until further use. A portion of the cardiac tissue was used for mRNA and protein expression assays; the remainder was fixed with 10% buffered formalin and embedded in paraffin for histology and immunofluorescence analysis.

### Experimental Procedures in T2DM Mice

We used C57BLKS/J Iar-+*lepr* *^db^*/+*lepr* *^db^* (*db/db*) mice for the T2DM animal model, and age-matched C57BLKS/J Iar-m+/+*lepr* *^db^* (*db/+*) mice were used as the control. Seven- to 8-week-old *db/db* mice were divided into three groups: (1) the T2DM group was treated with vehicle (0.1% DMSO, i.p., n=12), (2) the T2DM+CAR group was treated with carvacrol (20 mg/kg/day, i.p., n=12), and (3) the T2DM+ MET group was treated with metformin (100 mg/kg/day, i.p., n=12). Control mice were administered an equivalent volume of the vehicle. Six weeks after treatment, we measured body weight, random blood glucose, and fasting blood glucose and undertook echocardiography of the test animals. Thereafter, the mice were anesthetized and sacrificed as previously described. Cardiac tissues were collected for quantitative real-time PCR (qRT-PCR), Western blotting analysis, histology, and immunofluorescence analysis.

### Echocardiography

Cardiac function was evaluated by transthoracic echocardiography with a 25-MHz ultrasound transducer (Vevo 2100, VisualSonics). (Xiao et al., 2012) Sedation was induced with 2% isoflurane and maintained with 1–1.5% isoflurane in the mice. The parasternal long-axis view (B-mode and M-mode) was obtained, and measurements of cardiac structure and function were carried out. The thickness of the left ventricular posterior wall diameter (LVPW) and anterior left ventricular wall (LVAW) as well as left ventricular interior diameter (LVID) was measured from the left ventricle through M-mode tracing at the mid-papillary muscle level, and left ventricular ejection fraction (LVEF) and fractional shortening (LVFS) were calculated. Moreover, the passive left ventricular filling peak velocity (E, mm/s) and atrial contraction flow peak velocity (A, mm/s) were acquired from mitral valve Doppler flow images from an apical four-chamber view. Moreover, we calculated the ratio of early diastolic filling to atrial filling velocity of mitral flow (E/A ratio).

### Histology and Immunofluorescence Measurements

Murine hearts were fixed with 10% formalin and immersed in paraffin. For hematoxylin and eosin (H&E) and Masson’s trichrome staining, cardiac tissues from four mice in each group were embedded in paraffin and cut into 4-µm sections as previously described (Zhao et al., 2018). For the immunofluorescence assay (Hou et al., 2017), antigen retrieval was carried out in EDTA buffer (pH 9.0) by heating to 99°C for 20 min. After quenching the endogenous peroxidase with 3% H_2_O_2_, the sections were blocked with 10% non-immune goat serum (Life Technologies, Grand Island, NY, USA) and then incubated overnight with anti-GLUT4 (1:100, Novus Biologicals, CO, USA) at 4°C overnight. After washing with Tris-buffered saline containing 0.1% Tween 20 (TBST), the sections were co-incubated with secondary antibody conjugated with DyLight-555 and fluorescein isothiocyanate-conjugated wheat germ agglutinin (WGA-fluorescein; GeneTex, Inc., CA, USA). Nuclei were stained using 4’,6-diamidino-2-phenylindole (DAPI; Sigma-Aldrich, St. Louis, MO, USA). All confocal microscopic images were captured using a Nikon A1 confocal microscope (Nikon Instech Co., Ltd., Konan, Tokyo, Japan) under the same parameters. Sections from at least 5 samples of each group were stained, and 10 fields were randomly selected from each sample.

### RNA Isolation and qRT-PCR

Total RNA was prepared from the left ventricle of the heart using TRIzol (Life Technologies, Grand Island, NY, USA). Complementary DNA (cDNA) was synthesized using the First-Strand cDNA Synthesis Kit (Takara Bio Inc., Kusatsu, Shiga, Japan) in accordance with the manufacturer’s instructions. RT-qPCR was carried out in 20 µl system with the QuantiTect SYBR Green PCR Kit (Takara Bio Inc., Kusatsu, Shiga, Japan) on an ABI StepOneTM Real-Time PCR System (Thermo Fisher Scientific Inc., Waltham, MA, USA). The primer sequences used were as follows: natriuretic peptide A (*Nppa*), 5’-ACCCTGGGCTTCTTCCTCGTCTT-3’ (sense) and 5’-GCGGCCCCTGCTTCCTCA-3’ (anti-sense); β-myosin heavy chain (*Myh7*), 5’-GCCCTTTGACCTCAAGAAAG-3’ (sense) and 5’-CTTCACAGTCACCGTCTTG-3’ (anti-sense); and *Gapdh*, 5’-AGGTCGGTGTGAACGGATTTG-3’ (sense) and 5’-GGGGTCGTTGATGGCAACAA-3’ (antisense). *Gapdh* was used for normalization of the qPCR. The fold change in expression was calculated using the 2^−△△^CT method.

### Protein Extraction and Western Blotting Analysis

Membrane and cytoplasmic proteins were prepared using the Mem-PER^™^ Plus Membrane Protein Extraction Kit (Thermo Fisher Scientific, Rockford, IL, USA) as per the manufacturer’s instructions. RIPA lysis and extraction buffer supplemented with protease and phosphatase inhibitors were used for whole-tissue protein extraction. Protein concentrations were determined using the Pierce BCA Protein Assay Kit. Equal amounts of protein were separated by 8 or 10% sodium dodecyl sulfate polyacrylamide gel and transferred to polyvinylidene difluoride (PVDF) membranes. The PVDF membranes were blocked with 5% skim milk in TBST for 60 min at room temperature. The blot was incubated overnight with primary antibodies (**Table S1**) at 4°C. After being washed thrice in TBST, the PVDF membrane was incubated with a peroxidase-conjugated secondary antibody (Bioworld Technology Inc., MN, USA) at 1:5,000 dilution for 45 min at room temperature. The blot was developed by using the Pico Western blotting detection reagents according to the manufacturer’s instruction.

### Statistical Analysis

Data were expressed as mean ± SEM and analyzed by ANOVA in the SPSS18.0 software (IBM Corporation, Armonk, NY, USA). Levene’s tests for equal variances were conducted prior to one-way ANOVA. When equal variances were present, one-way ANOVA with Bonferroni *post hoc* test was used for multi-group comparisons. If equal variances were not assumed to be present, Tamhane’s T2 test was used for multiple comparison tests. *P*-value less than 0.05 was considered statistically significant.

## Results

### Effects of Carvacrol on Random Blood Glucose and Body Weight in Mice With T1DM

We investigated the effects of carvacrol on random blood glucose and body weight in mice with STZ-induced T1DM. In T1DM mice, carvacrol 20 mg/kg/day (i.p.) treatment for 4 weeks significantly reduced random blood glucose levels when compared with vehicle-only treatment (**Table 1**). Moreover, the glycemia-regulating effects of carvacrol were more obvious after 6 weeks of treatment—T1DM mice treated with carvacrol 10 or 20 mg/kg/day had significantly lower blood glucose levels than the control mice. The T1DM mice had significantly lower body weight than the control mice at the specified experimental time points; however, we did not find a significant between-group difference in the body weight of T1DM mice treated with carvacrol or vehicle only.

**Table 1:** Effects of carvacrol on random blood glucose and body weight in mice with STZ-induced type 1 diabetes mellitus.

Groups	Random blood glucose (mmol/L)			Body weight (g)		
	2 weeks	4 weeks	6 weeks	2 weeks	4 weeks	6 weeks
Con	9.38±0.16	7.81±0.31	8.67±0.94	27.71±0.11	28.67±0.53	30.75±1.11
Con20	9.39±0.44	8.04±0.75	9.16±0.48	27.28±0.19	27.86±0.69	29.71±0.95
T1DM	26.55±1.70**	27.21±0.93**	32.15±1.08**	22.38±0.72**	23.28±0.30**	22.25±0.70**
T1DM+CAR 10	25.48±1.33**	23.52±1.18**	20.70±1.47**^#^	22.03±0.89**	21.92±0.33**	20.89±0.44**
T1DM+CAR 20	24.04±0.88**	21.07±1.23**^#^	17.08±1.60**^##^	21.8±0.74**	21.41±0.39**	21.00±0.47**

Carvacrol was administered once daily after 4 weeks of diabetic induction. Random blood glucose levels and body weight were measured every 2 weeks after carvacrol administration. Con, control mice treated with vehicle (0.1% DMSO); Con20, control mice treated with 20 mg/kg/day of carvacrol; T1DM, STZ induced type 1 diabetic mice with vehicle treatment (0.1% DMSO); T1DM+CAR10, diabetic mice treated with 10 mg/kg/day carvacrol; T1DM+CAR20, diabetic mice treated with 20 mg/kg/day carvacrol. Values are means ± SEM from ten mice in each group. **P<0.01 vs. Con group; ^#^P<0.05 vs. T1DM group; ^##^P<0.01 vs. T1DM group.

### Carvacrol Attenuated Myocardial Remodeling in Mice With STZ-Induced T1DM

**Figure 2:**
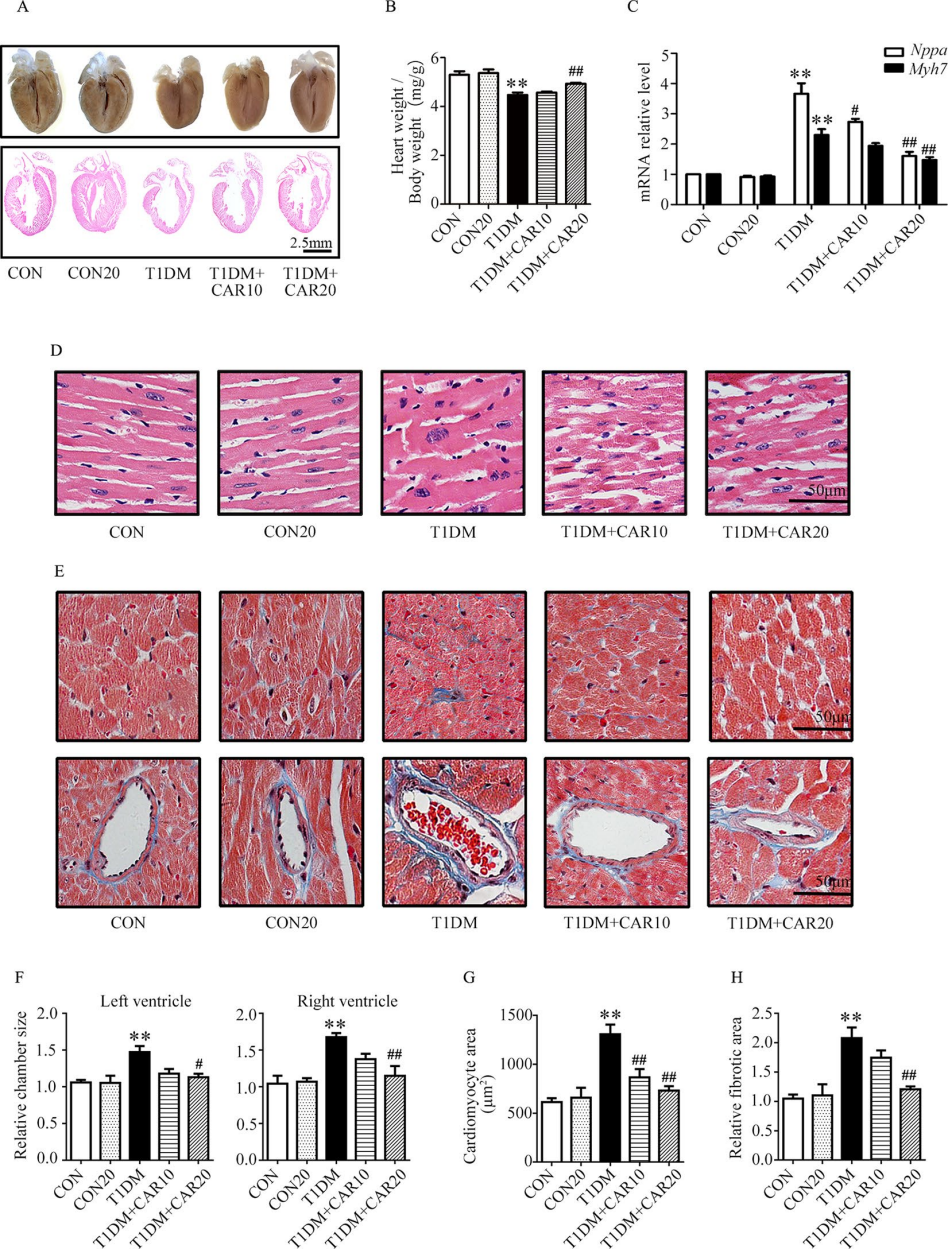
Carvacrol protects against hyperglycemia-induced cardiac remodeling in mice with type 1 diabetes mellitus (T1DM). **(A)** Gross view of whole hearts (upper); hematoxylin and eosin (H&E) staining of longitudinal sections of the heart in control mice or mice with STZ-induced T1DM after carvacrol or vehicle treatment for 6 weeks (lower); scale bar, 2.5 mm. **(B)** Heart weight/body weight (HW/BW) ratio of mice from the study groups. **(C)** Fold changes in natriuretic peptide A (*Nppa*, white rectangle) and β-myosin heavy chain (*Myh7*, black rectangle) mRNA levels determined by quantitative polymerase chain reaction (qPCR) in mouse hearts from the indicated study groups. **(D)** Representative images of H&E staining of control and diabetic mouse hearts after carvacrol or vehicle administration; scale bar, 50 μm. **(E)** Representative images of Masson’s trichrome staining for interstitial fibrosis of the myocardium (upper) and perivascular fibrosis (bottom) in five sets of mouse hearts from each group; scale bar, 50 μm. **(F)** Chamber sizes of left ventricle and right ventricle. **(G)** Quantification of cardiomyocytes area. **(H)** Quantification of relative fibrotic area. CON, control mice treated with vehicle (0.1% DMSO); CON20, control mice treated with 20 mg/kg/day carvacrol; T1DM, mice with STZ-induced type 1 diabetes mellitus with vehicle treatment (0.1% DMSO); T1DM+CAR10, diabetic mice treated with 10 mg/kg/day carvacrol; T1DM+CAR20, diabetic mice treated with 20 mg/kg/day carvacrol. Data are presented as mean ± SEM; 8–10 mice per group. **P < 0.01 *vs.* CON group; ^#^ *P* < 0.05 *vs.* T1DM group; ^##^ *P* < 0.01 *vs.* T1DM group.

Furthermore, we investigated the effects of carvacrol on cardiac hypertrophy and cardiac fibrosis in mice with T1DM. On examination of gross specimens and H&E-stained sagittal sections of murine hearts, we found decreased left ventricular wall thickness in T1DM mice as compared with the control mice; however, carvacrol 20 mg/kg/day prevented this morphologic change (**Figures 2A, F**). Meanwhile, mice with T1DM had a significantly smaller ratio of heart weight/body weight than the control mice; however, carvacrol 20 mg/kg/day significantly increased this ratio in mice with T1DM (**Figure 2B**). Furthermore, qPCR analysis showed that there was increased mRNA expression of two hypertrophic markers—*Nppa* and *Myh7*—in the hearts of mice with T1DM; carvacrol reduced the mRNA expression of both markers in a dose-dependent manner (**Figure 2C**). In addition, H&E staining of sagittal sections from left ventricular tissue showed that myocardial cells in the control mice lined up regularly, and carvacrol treatment did not affect their morphologic characteristics in the control mice (**Figures 2D, G**). However, in mice with T1DM, cardiomyocytes were hypertrophied and irregularly arranged (**Figures 2D, G**); nevertheless, the changes were attenuated by treatment with carvacrol (**Figures 2D, G**). Masson’s trichrome staining was undertaken to determine myocardial fibrosis. There was a marked increase in myocardial fibrosis in interstitial and perivascular areas in T1DM mice (**Figures 2E, H**), but this was significantly reduced by carvacrol treatment; this was more prominent with the 20 mg/kg/day carvacrol compared to the 10 mg/kg/day carvacrol treatment. Collectively, our results showed that carvacrol treatment attenuated cardiac remodeling in mice with STZ-induced T1DM.

### Carvacrol Alleviated Left Ventricular Dysfunction in Mice With STZ-Induced T1DM

**Figure 3:**
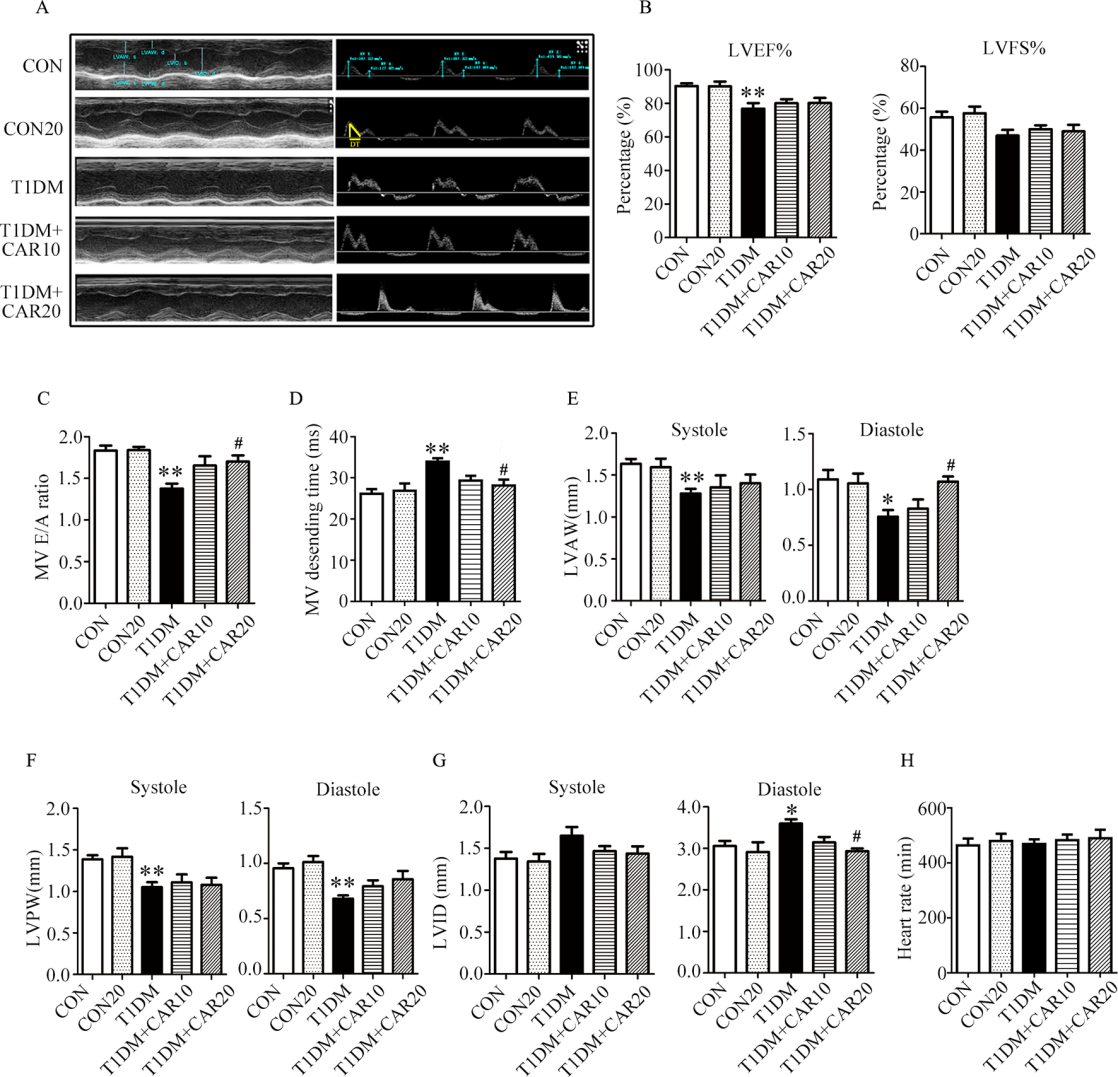
Echocardiographic examination indicated that carvacrol mitigated myocardial dysfunction in STZ-induced type 1 diabetic mice. **(A)** Representative M-mode images (left) and PW Doppler mode waveform images of mitral valve flow (right) in control mice or mice with STZ-induced T1DM of carvacrol or vehicle treatment for 6 weeks. **(B–H)** Cardiac parameters in the indicated groups were measured: left ventricular ejection fractions (LVEF%, **(B)** and left ventricular fractional shortening (LVFS%, **(B)**, early diastolic filling to atrial filling velocity ratio of mitral flow (E/A ratio, **(C)**, mitral valve descending time (DT, **D**), anterior left ventricular wall (LVAW, **E**), left ventricular posterior wall (LVPW, **F**), left ventricular internal diameter (LVID, **G**), and heart rate (HR, **H**). CON, control mice treated with vehicle (0.1% DMSO); CON20, control mice treated with 20 mg/kg/day carvacrol; T1DM, mice with STZ-induced type 1 diabetes mellitus with vehicle (0.1% DMSO); T1DM+CAR10, diabetic mice treated with 10 mg/kg/day carvacrol; T1DM+CAR20, diabetic mice treated with 20 mg/kg/day carvacrol. Data are presented as mean ± SEM of 8–10 mice per group. *P < 0.05 and **P < 0.01 *vs.* CON group; ^#^ *P* < 0.05, ^##^ *P* < 0.01 *vs.* T1DM group.

Mice with STZ-induced T1DM exhibited left ventricular dysfunction, manifested by decreased LVEF% on echocardiographic measurement (**Figures 3A, B**). Results of echocardiographic assessment showed that the LVAW and LVPW—in both end-systole and end-diastole—decreased, although LVID increased in end-diastole in mice with T1DM compared with control mice (**Figures 3E–G**); these findings agreed with the results of histological examination, which showed the LVAW thickness decreased in mice with T1DM (**Figures 3E–G**, *P* < 0.05). Using a pulsed wave Doppler technique, echocardiography revealed that diastolic cardiac function was significantly impaired in mice with STZ-induced diabetes (**Figures 3A, C, D**). The transmitral filling pattern showed an inverted E/A ratio (**Figures 3A, C**) with prolongation of the E-wave deceleration time (**Figure 3D**) in mice with STZ-induced diabetes, compared with the control group (*P* < 0.05). Carvacrol 20 mg/kg/day, but not 10 mg/kg/day, significantly increased the E/A ratio, decreased E-wave deceleration time, and increased LVAW in end-diastole, whereas it decreased the LVID in end-diastole, in mice with T1DM (**Figures 3C,–G**, *P* < 0.05). Heart rates were similar in the study groups (**Figure 3H**). Our results suggest that carvacrol improves cardiac diastolic function and cardiac remodeling in mice with STZ-induced T1DM.

### Effects of Carvacrol on Blood Glucose and Body Weight in Mice With T2DM

Next, we used *db/db* mice as an animal model of T2DM to elucidate the therapeutic effects of carvacrol on T2DM-induced DCM. Based on our results from mice with T1DM, where carvacrol 20 mg/kg/day significantly improved cardiac function, we administrated the same dosage of carvacrol to mice with T2DM. We used metformin as a positive drug control. The body weight of mice with T2DM significantly increased, as compared with control mice (*P* < 0.05; **Figure S1A**). Carvacrol significantly decreased body weight at 4 and 6 weeks after treatment, compared with the control, in mice with T2DM (*P* < 0.05). Furthermore, body weight significantly decreased 6 weeks after metformin treatment, compared with mice with T2DM (*P* < 0.05). Thus, both carvacrol and metformin treatments significantly decreased heart weight in mice with T2DM, compared with vehicle-treated mice with T2DM (**Figure S1B**; *P*<0.05). In addition, both carvacrol and metformin resulted in significant reduction of random and fasting blood glucose levels in mice with T2DM, compared with vehicle-treated mice with T2DM (**Figures S2A, B**; *P* < 0.05).

### Carvacrol Attenuated Myocardial Remodeling in Mice With T2DM

**Figure 4:**
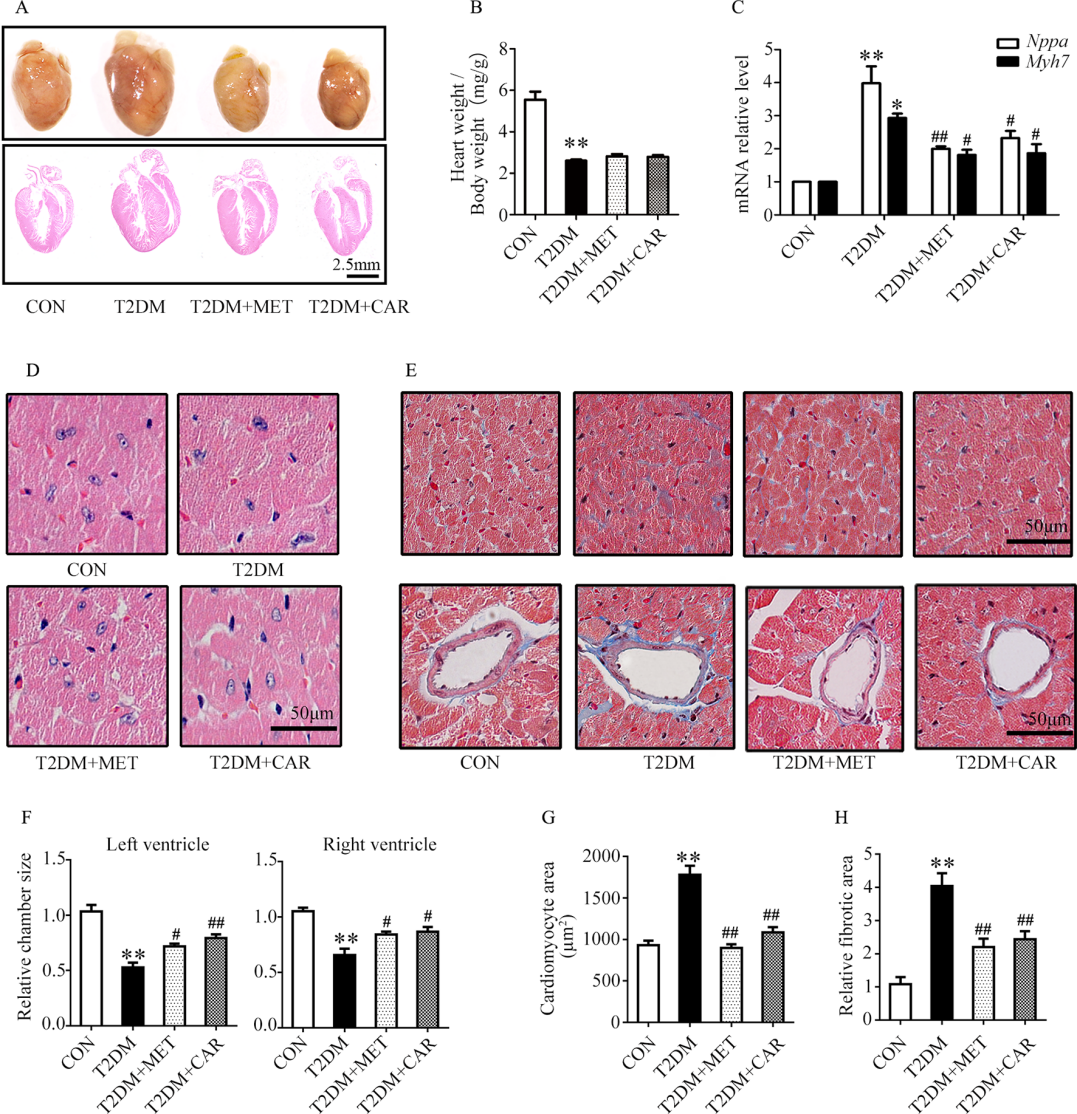
Carvacrol protected against hyperglycemia-induced cardiac remodeling in *db/db* mice. **(A)** Gross view of whole hearts (upper); hematoxylin and eosin (H&E) staining of longitudinal sections of the heart in control or *db/db* mice after carvacrol, metformin, or vehicle treatment for 6 weeks (bottom); scale bar, 2.5 mm. **(B)** Heart weight/body weight (HW/BW) ratio in mice from the indicated groups. **(C)** Fold changes in *Nppa* and *Myh7* mRNA levels, determined by qPCR, in murine hearts from the indicated groups. **(D)** Representative images of H&E staining of control and diabetic murine hearts after administration of carvacrol, metformin, or vehicle; scale bar, 50 μm. **(E)** Representative images of Masson’s trichrome staining for interstitial fibrosis of the myocardium (top) and perivascular fibrosis (bottom) in murine hearts from each group; scale bar, 50 μm. **(F)** Chamber sizes of the left and right ventricles. **(G)** Quantification of the area of cardiomyocytes. **(H)** Quantification of the relative fibrotic area. CON, control mice treated with vehicle (0.1% DMSO); T2DM, *db/db* mice treated with vehicle (0.1% DMSO); T2DM + MET, *db/db* mice treated with 100 mg/kg/day metformin; T2DM+CAR, *db/db* mice treated with 20 mg/kg/day carvacrol. Data are presented as mean ± SEM; 8–10 mice in each group. *P < 0.05, **P < 0.01 *vs.* CON group; ^#^ *P* < 0.05, ^##^ *P* < 0.01 *vs.* T2DM group.

Carvacrol exerted a similar effect as metformin, and both significantly attenuated cardiac hypertrophy in mice with T2DM as compared with the vehicle treatment (**Figure 4A**). The ratio of heart weight/body weight decreased in mice with T2DM, compared with control mice (**Figure 4B**, *P* < 0.05). There was no significant difference in the ratio of heart weight/body weight between mice with T2DM that were treated with carvacrol or metformin and those treated with vehicle only. Similar to the findings in mice with T1DM, the mRNA levels of the hypertrophic markers *Nppa* and *Myh7* significantly increased in the hearts of mice with T2DM in comparison with control mice (**Figure 4C**, *P* < 0.05); both carvacrol and metformin, compared with vehicle-only treatment, markedly reduced the mRNA expression of these two hypertrophic markers in the mice with T2DM (**Figure 4C**, *P* < 0.05). In addition, H&E-staining showed that, compared with vehicle, carvacrol and metformin treatments markedly attenuated morphologic alterations in cardiac tissue that manifested as decreased cross-sectional area of cardiomyocytes in mice with T2DM (**Figures 4D, F, G**). Meanwhile, Masson’s trichrome staining showed that there was an observable increase in myocardial fibrosis in the heart of mice with T2DM as compared with control mice; however, myocardial fibrosis was significantly reduced by carvacrol or metformin treatment (**Figures 4E, H**, *P* < 0.05). These results demonstrate that carvacrol could attenuate myocardial remodeling besides its hypoglycemic effect in mice with T2DM.

### Carvacrol Improved Left Ventricular Function in Mice With T2DM

**Figure 5:**
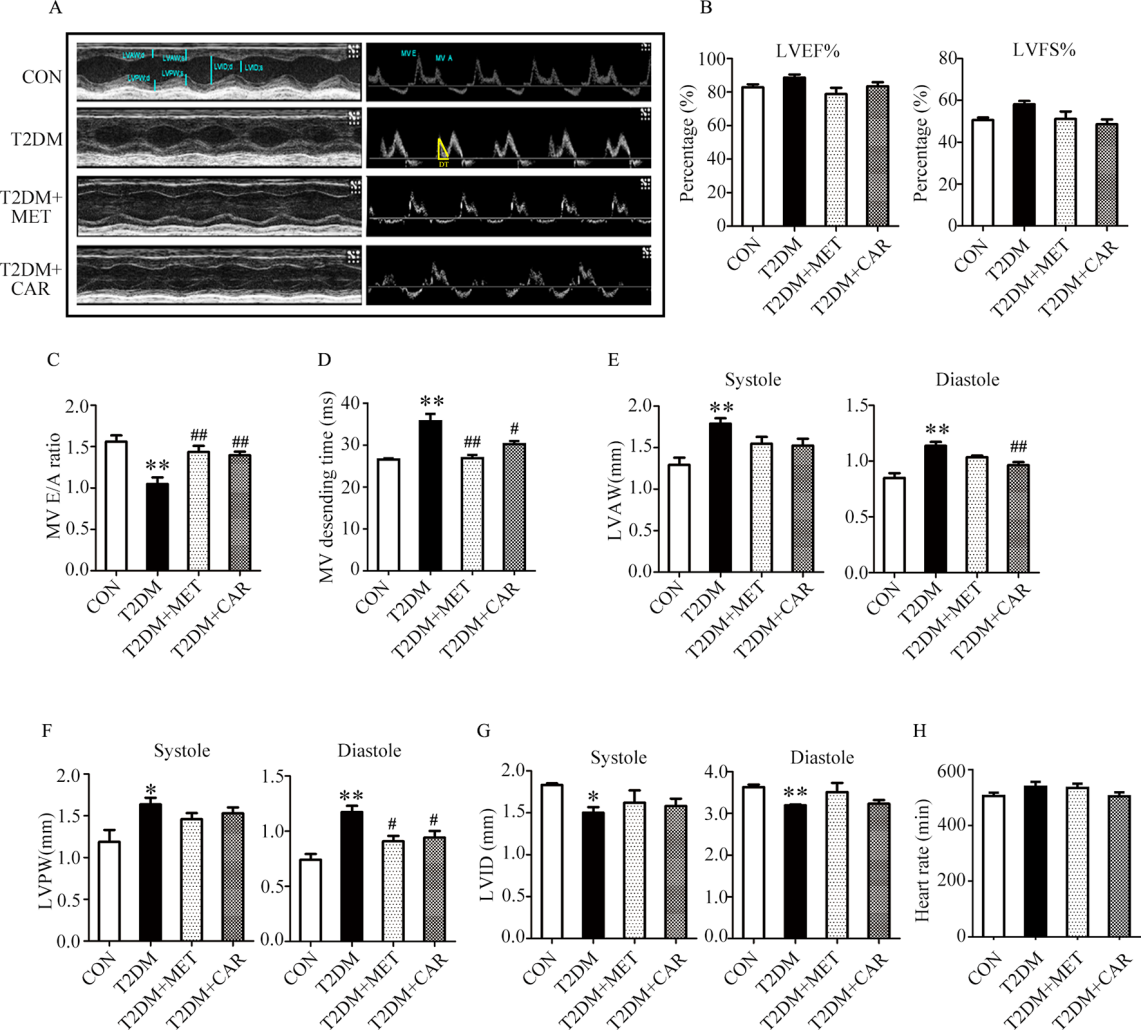
Carvacrol improved myocardial dysfunction, as indicated by echocardiographic examination in *db/db* mice. **(A)** Representative M-mode images (left) and PW Doppler mode waveform images of mitral valve flow (right) in control mice or diabetic *db/db* mice after treatment with carvacrol, metformin, or vehicle for 6 weeks. **(B**–**H)** Cardiac parameters were measured in the indicated groups and included: left ventricular ejection fractions (LVEF%, **B**) and left ventricular fractional shortening (LVFS%, **B**), ratio of early diastolic filling to atrial filling velocity of mitral flow (E/A ratio, **C**), mitral valve descending time (DT, **D**), anterior left ventricular wall (LVAW, **E**), left ventricular posterior wall (LVPW, **F**), left ventricular internal diameter (LVID, **G**), and heart rate (HR, **H**). CON, control mice treated with vehicle (0.1% DMSO); T2DM, *db/db* mice treated with vehicle (0.1% DMSO); T2DM+MET, *db/db* mice treated with 100 mg/kg/day metformin; T2DM+CAR, *db/db* mice treated with 20 mg/kg/day carvacrol. Data are presented as means ± SEM; 8–10 mice in each group. *P < 0.05, **P < 0.01 *vs.* CON group; ^#^ *P* < 0.05, ^##^ *P* < 0.01 *vs.* T2DM group.

Mice with T2DM developed left ventricular dysfunction and diastolic cardiac dysfunction at the end of the experimental period (**Figures 5A–H**). In both end-systole and end-diastole, these mice showed significant decreases in the E/A ratio and LVID as well as significant increases in E-wave deceleration time, LVAW, and LVPW when compared with control mice (**Figures 5C–G**, *P* < 0.05). However, carvacrol treatment significantly increased the E/A ratio and decreased both LVAW and LVPW in end-diastole as well as the E-wave deceleration time in mice with T2DM, compared with mice that received vehicle-only treatment (*P* < 0.05). Similarly, metformin increased the E/A ratio and reduced the LVPW in end-diastole in mice with T2DM as compared to treatment with the vehicle ( **Figures 5C–F**, *P* < 0.05). We found no significant between-group differences in LVEF%, LVFS%, and heart rate for all study groups (**Figures 5B, H**). These results showed carvacrol attenuated cardiac hypertrophy and improved diastolic function in mice with T2DM.

### Effects of Carvacrol Treatment on the PI3K/AKT Signaling Pathway in the Hearts of Mice With T1DM and T2DM

**Figure 6:**
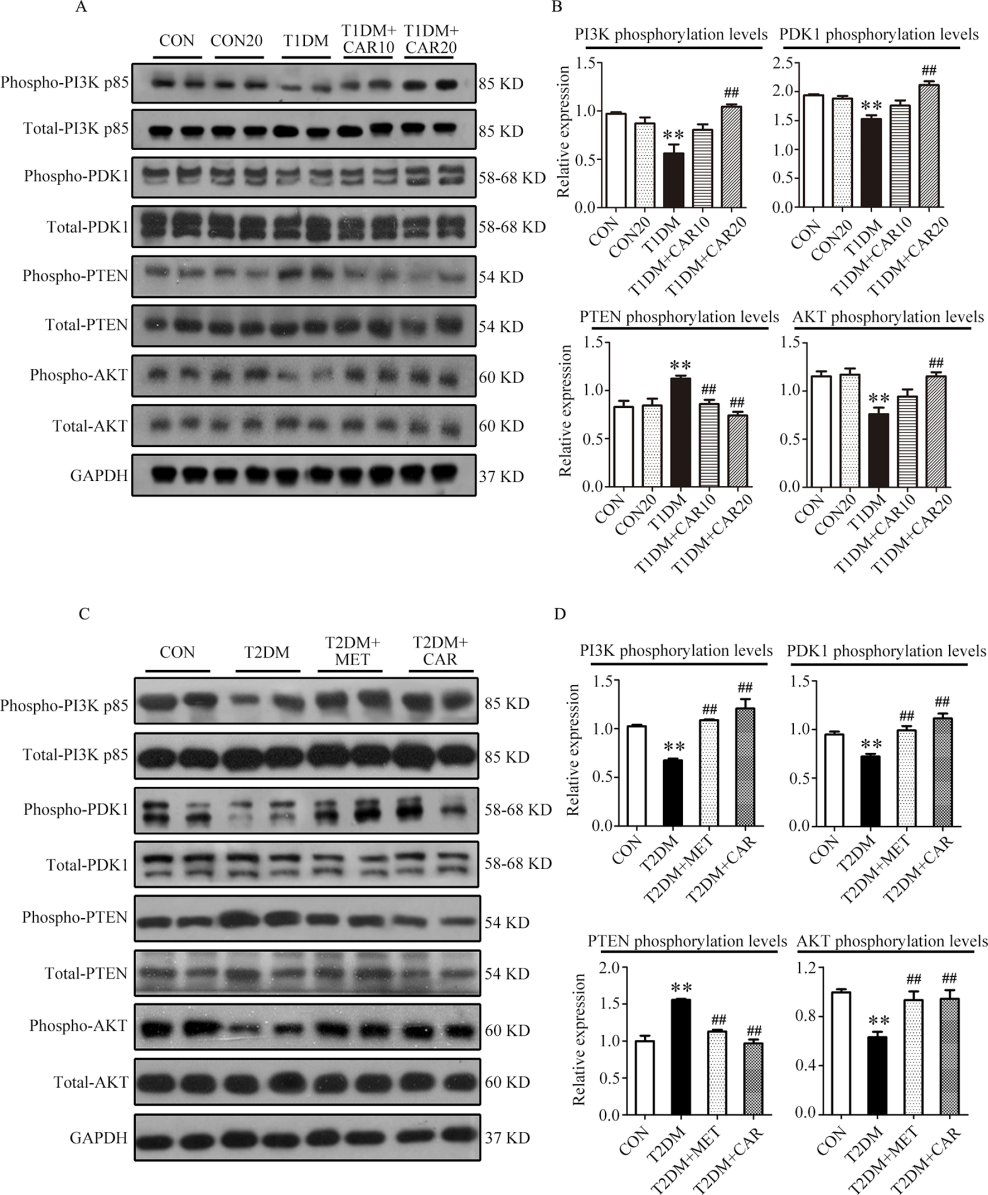
Carvacrol attenuates diabetic myocardial remodeling by restoring the PI3K/AKT pathway in diabetic mice. **(A** and **B)** Representative Western blotting images of whole lysate **(A)** and fold changes **(B)** in relative densitometric values of p-PI3K p85, p-PDK1, p-PTEN, and p-AKT in hearts from mice with STZ-induced type 1 diabetes treated with 10 mg/kg/day carvacrol, 20 mg/kg/day carvacrol, or vehicle. CON, control mice treated with vehicle (0.1% DMSO); CON20, control mice treated with 20 mg/kg/day carvacrol; T1DM, mice with STZ-induced type 1 diabetes mellitus with vehicle (0.1% DMSO); T1DM+CAR10, diabetic mice treated with 10 mg/kg/day carvacrol; T1DM+CAR20, diabetic mice treated with 20 mg/kg/day carvacrol. Data are presented as means ± SEM; 8–10 mice per group. *P < 0.05 and **P < 0.01 *vs.* CON group; ^#^ *P* < 0.05, ^##^ *P* < 0.01 *vs*. T1DM group. **(C** and **D)** Representative Western blotting images of whole lysate **(A)** and fold changes **(B)** in relative densitometric values of phospho-PI3K p85, p-PDK1, p-PTEN, and p-AKT in murine hearts from the indicated groups. CON, control mice treated with vehicle (0.1% DMSO); T2DM, *db/db* mice treated with vehicle (0.1% DMSO); T2DM+MET, *db/db* mice treated with 100 mg/kg/day metformin; T2DM+CAR, *db/db* mice treated with 20 mg/kg/day carvacrol. Data are presented as means ± SEM of 8–10 mice in each group. *P < 0.05, **P < 0.01 *vs*, CON group; ^#^ *P* < 0.05, ^##^ *P* < 0.01 *vs*. T2DM group.

Compared with the control mice, the mice with STZ-induced T1DM showed significant suppression of PI3K/AKT signaling in the hypertrophic heart, where phosphorylation of PI3K, PDK1, and AKT was significantly reduced and that of PTEN was significantly increased (**Figures 6A, B**, *P* < 0.05). Carvacrol (20 mg/kg/day) treatment significantly inhibited the changes of these signal proteins of the PI3K/AKT signaling pathway in the heart of mice with T1DM, compared with the vehicle-only treatment (**Figures 6A, B**, *P* < 0.05). Furthermore, phosphorylation of PI3K, PDK1, and AKT significantly decreased consistently in the heart of mice with T2DM as compared with the control mice (**Figures 6C, D**, *P* < 0.05). Both carvacrol and metformin treatments significantly promoted the phosphorylation of PI3K, PDK1, and AKT and significantly reduced the expression of PTEN phosphorylation in mice with T2DM compared with vehicle treatment (*P* < 0.05). Thus, carvacrol exerted anti-DCM effects in both mice with T1DM and T2DM, potentially through the PI3K/AKT signaling pathway.

### Carvacrol Strengthened GLUT4 Membrane Translocation in Cardiomyocytes of Diabetic Mice

**Figure 7:**
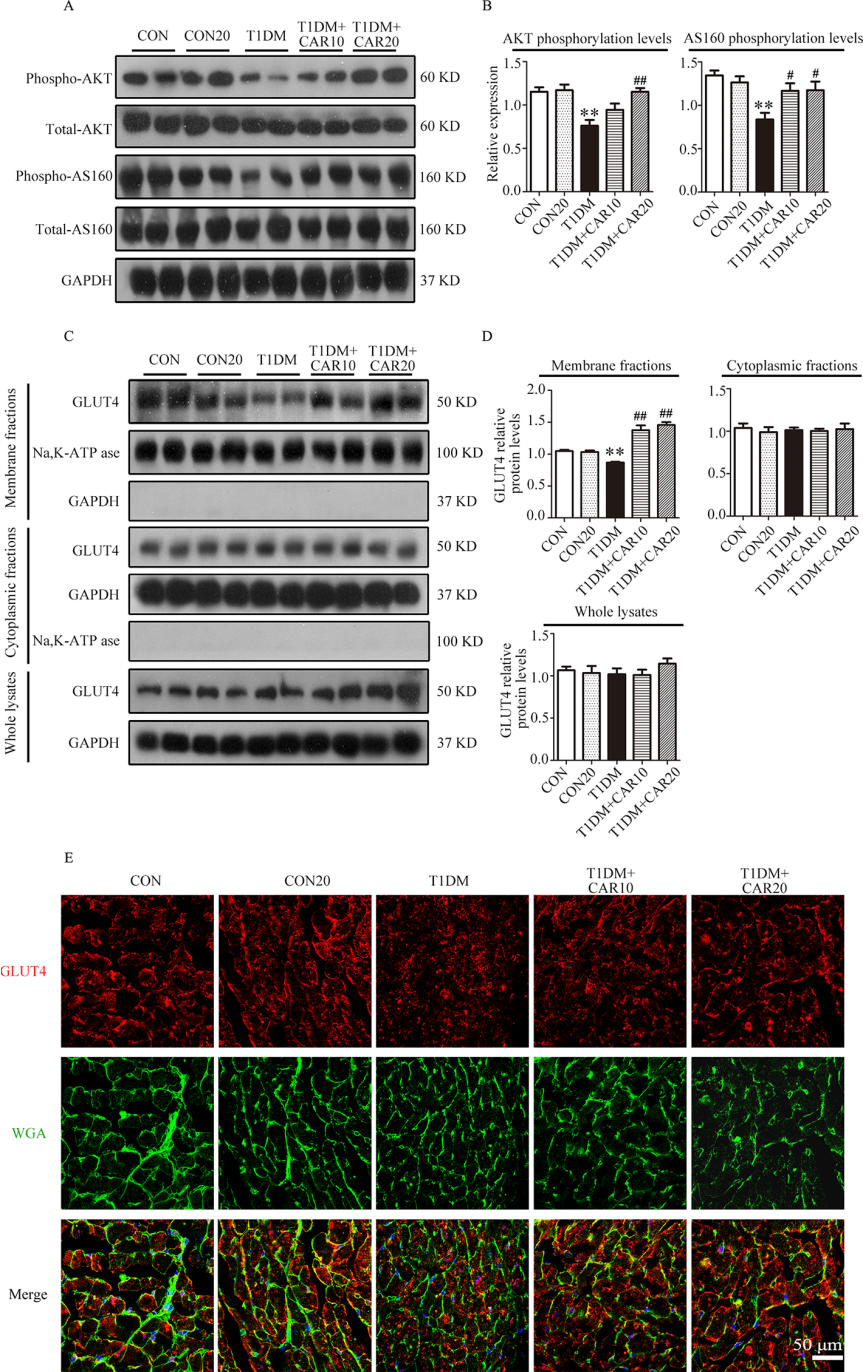
Carvacrol stimulated GLUT4 translocation by activating the AKT/AS160 pathway in the hearts of mice with STZ-induced diabetes. **(A** and **B)** Representative Western blotting images of whole lysate **(A)** and fold changes **(B)** in relative densitometric values of p-AKT and p-AS160 in the hearts of mice with STZ-induced type 1 diabetes treated with 10 mg/kg/day carvacrol, 20 mg/kg/day carvacrol, or vehicle. **(C** and **D)** Representative Western blotting images **(C)** and fold changes **(D)** of GLUT4 protein levels by relative densitometric values in membrane, cytoplasm, and whole lysates from murine hearts of the indicated groups. **(E)** Representative confocal microscopy of triple stains of GLUT4 (red), wheat germ agglutinin (WGA, green), and 4’,6-diamidino-2-phenylindole (DAPI, blue) in murine hearts of the indicated groups. Representative images from five sets of staining are shown. CON, control mice treated with vehicle (0.1% DMSO); CON20, control mice treated with 20 mg/kg/day carvacrol; T1DM, mice with STZ-induced type 1 diabetes mellitus with vehicle treatment (0.1% DMSO); T1DM+CAR10, diabetic mice treated with 10 mg/kg/day carvacrol; T1DM+CAR20, diabetic mice treated with 20 mg/kg/day carvacrol. Data are presented as the means ± SEM of 8–10 mice per group. *P < 0.05 and **P < 0.01 *vs*. CON group; ^#^ *P* < 0.05, ^##^ *P* < 0.01 *vs.* T1DM group.

PI3K/AKT signaling promotes GLUT4 recruitment to the cell membrane and thereby enhances glucose uptake in cardiac tissues (Szablewski, 2017). Next, we evaluated whether carvacrol regulates GLUT4 function. Western blotting showed that levels of phosphorylated AKT and AKT substrates (molecular weight 160 kDa; AS160; **Figures 7A, B**) significantly decreased in the heart of mice with T1DM when compared with control mice; carvacrol treatment restored the levels of phosphorylated AKT and AS160. Furthermore, GLUT4 translocation to plasma membrane was significantly reduced in cardiac tissues of mice with T1DM when compared with control mice (**Figures 7C, D**, *P* < 0.05). In contrast, carvacrol treatment significantly enhanced GLUT4 membrane translocation in T1DM mice, when compared with vehicle treatment (**Figures 7C, D**, *P* < 0.05). We carried out Immunofluorescent labeling to confirm the results of Western blotting for GLUT4 translocation. With WGA as the plasma membrane marker, we double stained GLUT4 with WGA (**Figure 7E**). Carvacrol treatment appeared to increase GLUT4 translocation to the plasma membrane.

**Figure 8:**
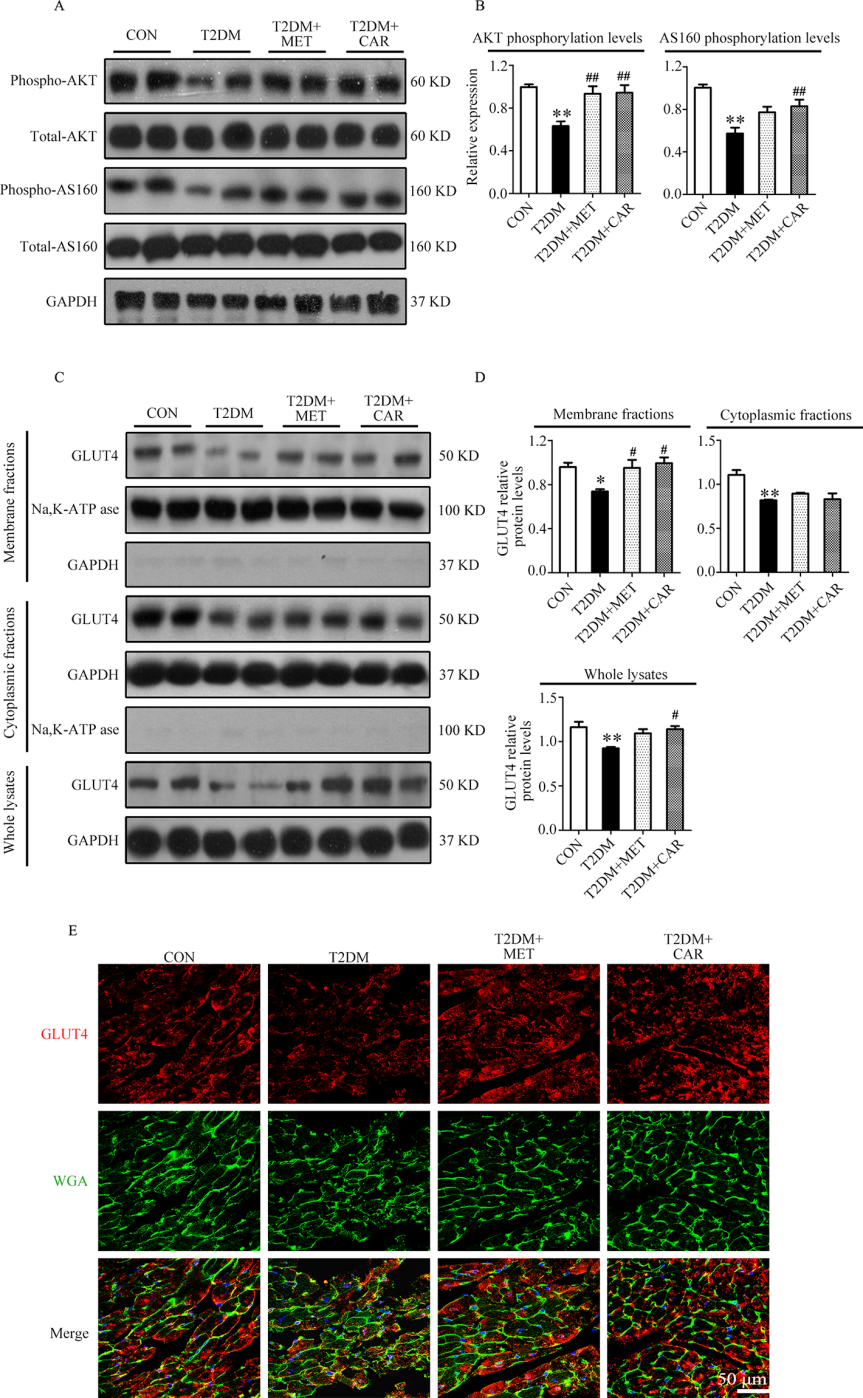
Carvacrol enhanced membrane translocation of GLUT4 by upregulating the AKT/AS160 pathway in the hearts of *db/db* mice. **(A** and **B)** Representative Western blotting images of whole lysate **(A)** and fold changes **(B)** in relative densitometric values of p-AKT and p-AS160 in murine hearts from the indicated groups. **(C** and **D)** Representative Western blotting images **(C)** and fold changes **(D)** of GLUT4 protein levels by relative densitometric values in membrane, cytoplasm, and whole lysates from murine hearts in each study group. **(E)** Representative confocal images of triple stains of GLUT4 (red), wheat germ agglutinin (WGA, green), and 4’,6-diamidino-2-phenylindole (DAPI, blue) in the murine hearts of the indicated groups. Representative images from five sets of staining are shown. CON, control mice treated with vehicle (0.1% DMSO); T2DM, *db/db* mice treated with vehicle (0.1% DMSO); T2DM+MET, *db/db* mice treated with 100 mg/kg/day metformin; T2DM+CAR, *db/db* mice treated with 20 mg/kg/day carvacrol. Data are presented as means ± SEM; 8–10 mice in each group. *P < 0.05, **P < 0.01 *vs.* CON group; ^#^ *P* < 0.05, ^##^ *P* < 0.01 *vs.* T2DM group.

Similar to the results in mice with T1DM, we found that AKT and AS160 phosphorylations as well as GLUT4 translocation to the plasma membrane were significantly reduced in cardiac tissues of mice with T2DM in comparison with control mice (*P* < 0.05); however, these changes reverted on carvacrol treatment (**Figures 8A–D**, *P* < 0.05). Moreover, metformin significantly restored AKT phosphorylation and GLUT4 translocation to the plasma membrane in the heart of mice with T2DM (*P* < 0.05); however, metformin did not significantly increase AS160 phosphorylation (*P* > 0.05). Double staining of GLUT4 and WGA further supported the results from Western blotting—that both carvacrol and metformin treatments promoted GLUT4 translocation to the plasma membrane in mice with T2DM (**Figure 8E**).

## Discussion

This study was conducted to investigate the effects of carvacrol on DCM in both mice with T1DM and those with T2DM. We confirmed the glycemia-regulating effects of carvacrol. Carvacrol protected against DM-induced cardiac remodeling and alleviated myocardial dysfunction. In addition, we showed that the PI3K/AKT signaling pathway is impaired in the heart of both mice with T1DM and T2DM; however, administration of carvacrol could restore the PI3K/AKT signaling pathway by increasing the levels of phosphorylated PI3K, PDK1, and AKT and reducing PTEN phosphorylation. Moreover, carvacrol significantly promoted GLUT4 membrane translocation and increased phosphorylation of AS160 in the hearts of both mice with T1DM and those with T2DM.

DCM is a common complication of DM. Early-stage DCM has diverse pathological changes, including left ventricular hypertrophy and cardiac fibrosis, which are associated with cardiac remodeling (Murtaza et al., 2019). These pathological changes cause cardiac stiffness and consequent left ventricular diastole (Murtaza et al., 2019). The present study validated our earlier finding (Yuan et al., 2016) that diabetes leads to cardiac remodeling and diastolic dysfunction in both mice with T1DM and T2DM; this finding was supported by the increased gene expression of cardiac hypertrophic markers (*Nppa* and *Myh7*), aggravation of cardiac fibrosis, and decreased E/A ratio in both types of diabetic mice. Furthermore, these changes indicate that mice with T1DM and T2DM had DCM, which significantly improved on carvacrol treatment. To the best of our knowledge, this is the first report of the protective effects of carvacrol against DCM. Although both T1DM and T2DM can cause DCM in terms of cardiac remodeling and impairment of left ventricular function (Murtaza et al., 2019), the T2DM-induced cardiac hypertrophy is likely different from that induced by T1DM. In general, T2DM is associated with a significant increase of heart weight, septal thickness, and increased thickness of the LVAW, whereas histopathological cardiac changes in T1DM are heterogeneous (Fuentes-Antras et al., 2015). T1DM could induce progressive cardiac concentric hypertrophy similar to that caused by T2DM; however, it caused extensive ventricular dilatational remodeling that was characterized by a reduction of the LVAW thickness and an increase of LVID (Nemoto et al., 2006). In addition, H&E staining of left ventricular tissue and echocardiography showed that there was thinning of the LVAW in mice with T1DM, compared to control mice; the LVID in T1DM mice increased, thereby demonstrating that mice with STZ-induced T1DM had extensive ventricular dilatational hypertrophy. In contrast to mice with T1DM, mice with T2DM showed increased heart weight and LVAW thickness as well as decreased LVID. Interestingly, although T1DM and T2DM lead to different types of cardiac remodeling, carvacrol could improve the cardiac morphologic changes caused by both types of diabetes. This indicates carvacrol protects against DCM of both T1DM and T2DM, potentially through the regulation of common signaling pathways.

Complex metabolic pathways, such as hyperglycemia and abnormal insulin signaling, are involved in DCM pathogenesis (Jia et al., 2018). Chronic hyperglycemia can lead to increased production of AGEs, which contributes to cardiac remodeling (Jia et al., 2016). Carvacrol reduces serum glucose levels in rats with STZ-induced T1DM and exerts antihyperglycemic effects in mice with high-fat diet–induced T2DM (Bayramoglu et al., 2014; Ezhumalai et al., 2014). We validated the antihyperglycemic effects of carvacrol in mice with STZ-induced T1DM mice and *db/db* mice with T2DM, which are potentially beneficial for cardiac structure and function in diabetes. However, the molecular signaling pathway involved in the glycemia-regulatory effects of carvacrol remains undefined. The recruitment of GLUT4 to the plasma membrane is essential for glucose uptake into cardiac tissues to maintain normal energy supply to the heart. Decreased protein expression and translocation of GLUT4 to the cell surface in cardiomyocytes have been found in both T1DM and T2DM (Szablewski, 2017); restoration of GLUT4 function was shown to suppress DCM progression (Zhao et al., 2018). We found that carvacrol significantly enhanced GLUT4 translocation to the cell membrane of cardiomyocytes in both mice with T1DM and those with T2DM. Our data suggest that upregulation of membrane translocation of GLUT4, which is commonly impaired in both T1DM and T2DM, is a potential mechanism underlying carvacrol-mediated improvement in cardiac glucose utilization, simultaneously as its blood glucose-lowering effect, and attenuation of left ventricular function in both types of diabetes.

**Figure 9:**
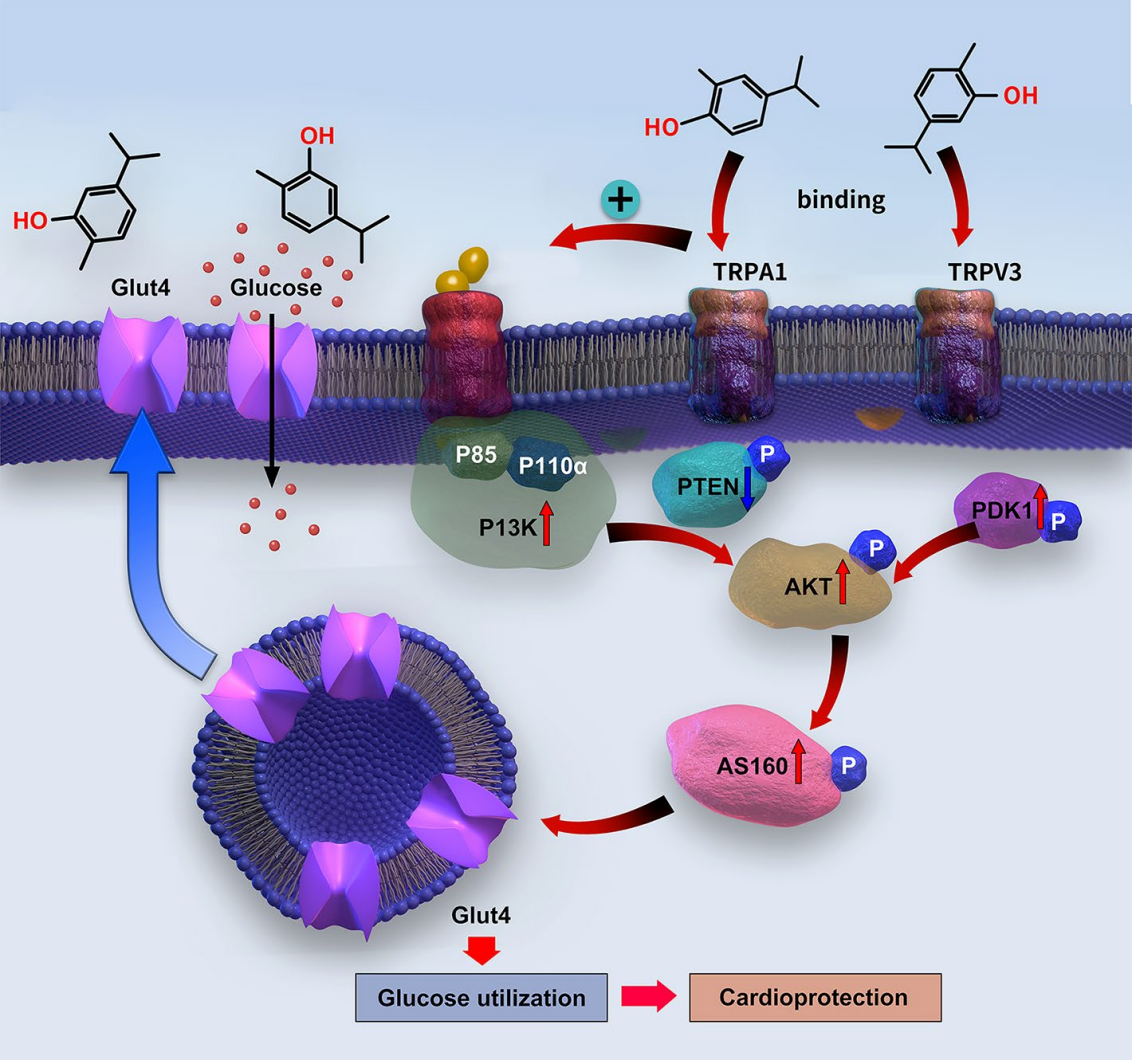
Diagram of the signaling mechanism involved in the myocardial protection mediated by carvacrol.

The PI3K/AKT pathway works as an upstream signaling route to stimulate GLUT4 translocation to the cell surface in cardiomyocytes, thereby promoting glucose uptake (Jia et al., 2018). GLUT4 translocation is mediated by AS160, which is activated through AKT phosphorylation (Kim et al., 2011). The PI3K protein consists of a p110 binding subunit and p85 regulatory subunit (Oudit and Penninger, 2009). Under physiological conditions, insulin activates PI3K *via* the insulin receptor substrate-1 to promote its main downstream molecular proteins—AKT and PDK1—which mediate C-terminal domain binding to phosphatidylinositol (3,4,5)-trisphosphate (PIP3) (Yao et al., 2014). AKT is activated through PI3K phosphorylation on Ser472 and Thr308, whereas PDK1 phosphorylates AKT on Thr308. In contrast, PTEN can convert PIP3 into PIP2, thereby downregulating AKT activity (Yao et al., 2014). The insulin shortage and insulin resistance in T1DM and T2DM, respectively, lead to suppression of PI3K/AKT signaling (Jia et al., 2018) and, consequently, to cardiac dysfunction. However, promoting the activation of PI3K/AKT can improve cardiac function (Siddall et al., 2008). In the present study, carvacrol simultaneously and dose-dependently enhanced membrane translocation of GLUT4; restored the phosphorylation of PI3K, PDK1, AKT, and AS160; and reduced the PTEN phosphorylation in the hearts of mice with T1DM. Carvacrol had similar effects on PI3K/AKT/GLUT4 signaling in T2DM and T1DM mice, wherein it activated PI3K/AKT signaling and increased GLUT4 membrane translocation. We used metformin as the positive control drug. Carvacrol had similar effects as metformin in reduction of blood glucose, suppression of cardiac remodeling, restoration of PI3K/AKT signaling, and promotion of GLUT4 membrane translocation. Our results indicate carvacrol significantly activates PI3K/AKT signaling in both T1DM and T2DM by increasing the phosphorylation of PI3K, AKT, PDK1, and AS160; reducing the phosphorylation of PTEN; and subsequently facilitating GLUT4 membrane translocation (**Figure 9**).

Carvacrol is one of the most abundant chemical constituents in the oil of oregano, which has widespread utility for health issues in the Mediterranean regions for centuries. This longstanding use suggests that carvacrol has a good safety profile for *in vivo* usage (Sharifi-Rad et al., 2018). Therefore, the clinical application of carvacrol appears feasible, which subject to further clinical validation of its therapeutic effects on DCM in patients with diabetes.

In summary, our study demonstrated that carvacrol protects against T1DM- and T2DM-induced cardiac remodeling and improves left ventricular function, potentially through promotion of PI3K/AKT signaling-mediated GLUT4 translocation to the plasma membrane that enhances glucose uptake by cardiomyocytes. Our findings indicate that carvacrol could be a promising therapeutic compound for the treatment of DCM.

## Data Availability

The datasets generated for this study are available on request to the corresponding author.

## Ethics Statement

The animal study was reviewed and approved by the ethics committee of Guangzhou Medical University.

## Author Contributions

NH, YPM, XXQ, WCY, and J-dL participated in research design. NH, YPM, XXQ, WCY, YLL, and GPZ conducted experiments. NH and J-dL supervised experiments. CFL, YL, and GPZ contributed new reagents or analytic tools. NH, YPM, XXQ, and GJZ performed data analysis. NH, YPM, WCY, and J-dL wrote or contributed to the writing of the manuscript.

## Conflict of Interest Statement

The authors declare that the research was conducted in the absence of any commercial or financial relationships that could be construed as a potential conflict of interest.

## References

[B1] Bayramoglu G. Senturk H. Bayramoglu A. Uyanoglu M. Colak S. Ozmen A. (2014). Carvacrol partially reverses symptoms of diabetes in STZ-induced diabetic rats. Cytotechnology 66 (2), 251–257. 10.1007/s10616-013-9563-5 23579248 PMC3918264

[B2] Camps M. Castello A. Munoz P. Monfar M. Testar X. Palacin M. (1992). Effect of diabetes and fasting on GLUT-4 (muscle/fat) glucose-transporter expression in insulin-sensitive tissues. Heterogeneous response in heart, red and white muscle. Biochem. J. 282 (Pt 3), 765–772. 10.1042/bj2820765 1554359 PMC1130854

[B3] Chenet A. L. Duarte A. R. de Almeida F. J. S. Andrade C. M. B. de Oliveira M. R. (2019). Carvacrol depends on heme oxygenase-1 (HO-1) to exert antioxidant, anti-inflammatory, and mitochondria-related protection in the human neuroblastoma SH-SY5Y cells line exposed to hydrogen peroxide. Neurochem. Res. 44 (4), 884–896. 10.1007/s11064-019-02724-5 30652259

[B4] Dandamudi S. Slusser J. Mahoney D. W. Redfield M. M. Rodeheffer R. J. Chen H. H. (2014). The prevalence of diabetic cardiomyopathy: a population-based study in Olmsted County, Minnesota. J. Card. Fail. 20 (5), 304–309. 10.1016/j.cardfail.2014.02.007 24576788 PMC4076144

[B5] Deng W. Lu H. Teng J. (2013). Carvacrol attenuates diabetes-associated cognitive deficits in rats. J. Mol. Neurosci. 51 (3), 813–819. 10.1007/s12031-013-0069-6 23877802

[B6] Desrois M. Sidell R. J. Gauguier D. King L. M. Radda G. K. Clarke K. (2004). Initial steps of insulin signaling and glucose transport are defective in the type 2 diabetic rat heart. Cardiovasc. Res. 61 (2), 288–296. 10.1016/j.cardiores.2003.11.021 14736545

[B7] Ezhumalai M. Radhiga T. Pugalendi K. V. (2014). Antihyperglycemic effect of carvacrol in combination with rosiglitazone in high-fat diet-induced type 2 diabetic C57BL/6J mice. Mol. Cell. Biochem. 385 (1–2), 23–31. 10.1007/s11010-013-1810-8 24057121

[B8] Fuentes-Antras J. Picatoste B. Gomez-Hernandez A. Egido J. Tunon J. Lorenzo O. (2015). Updating experimental models of diabetic cardiomyopathy. J. Diabetes Res. 2015, 656795. 10.1155/2015/656795 25973429 PMC4417999

[B9] Hou N. Wen Y. Yuan X. Xu H. Wang X. Li F. (2017). Activation of Yap1/Taz signaling in ischemic heart disease and dilated cardiomyopathy. Exp. Mol. Pathol. 103 (3), 267–275. 10.1016/j.yexmp.2017.11.006 29154888 PMC5988229

[B10] Huang J. P. Huang S. S. Deng J. Y. Hung L. M. (2009). Impairment of insulin-stimulated Akt/GLUT4 signaling is associated with cardiac contractile dysfunction and aggravates I/R injury in STZ-diabetic rats. J. Biomed. Sci. 16, 77. 10.1186/1423-0127-16-77 19706162 PMC2740847

[B11] Jia G. DeMarco V. G. Sowers J. R. (2016). Insulin resistance and hyperinsulinaemia in diabetic cardiomyopathy. Nat. Rev. Endocrinol. 12 (3), 144–153. 10.1038/nrendo.2015.216 26678809 PMC4753054

[B12] Jia G. Whaley-Connell A. Sowers J. R. (2018). Diabetic cardiomyopathy: a hyperglycaemia- and insulin-resistance-induced heart disease. Diabetologia 61 (1), 21–28. 10.1007/s00125-017-4390-4 28776083 PMC5720913

[B13] Kim H. Y. Choi H. J. Lim J. S. Park E. J. Jung H. J. Lee Y. J. (2011). Emerging role of Akt substrate protein AS160 in the regulation of AQP2 translocation. Am. J. Physiol. Renal Physiol. 301 (1), F151–F161. 10.1152/ajprenal.00519.2010 21511697

[B14] Kohn A. D. Summers S. A. Birnbaum M. J. Roth R. A. (1996). Expression of a constitutively active Akt Ser/Thr kinase in 3T3-L1 adipocytes stimulates glucose uptake and glucose transporter 4 translocation. J. Biol. Chem. 271 (49), 31372–31378. 10.1074/jbc.271.49.31372 8940145

[B15] Mbese Z. Aderibigbe B. A. (2018). Biological efficacy of carvacrol analogues. Recent Pat. Antiinfect. Drug Discov. 13 (3), 207–216. 10.2174/1574891X14666181205111821 30516115

[B16] Mueckler M. Thorens B. (2013). The SLC2 (GLUT) family of membrane transporters. Mol. Aspects Med. 34 (2–3), 121–138. 10.1016/j.mam.2012.07.001 23506862 PMC4104978

[B17] Murtaza G. Virk H. U. H. Khalid M. Lavie C. J. Ventura H. Mukherjee D. (2019). Diabetic cardiomyopathy—a comprehensive updated review. Prog. Cardiovasc. Dis. 10.1016/j.pcad.2019.03.003 30922976

[B18] Nemoto O. Kawaguchi M. Yaoita H. Miyake K. Maehara K. Maruyama Y. (2006). Left ventricular dysfunction and remodeling in streptozotocin-induced diabetic rats. Circ. J. 70 (3), 327–334. 10.1253/circj.70.327 16501301

[B19] Oudit G. Y. Penninger J. M. (2009). Cardiac regulation by phosphoinositide 3-kinases and PTEN. Cardiovasc. Res. 82 (2), 250–260. 10.1093/cvr/cvp014 19147653

[B20] Parnas M. Peters M. Dadon D. Lev S. Vertkin I. Slutsky I. (2009). Carvacrol is a novel inhibitor of Drosophila TRPL and mammalian TRPM7 channels. Cell Calcium 45 (3), 300–309. 10.1016/j.ceca.2008.11.009 19135721 PMC2680423

[B21] Qi K. Zhong J. (2018). LncRNA HOTAIR improves diabetic cardiomyopathy by increasing viability of cardiomyocytes through activation of the PI3K/Akt pathway. Exp. Ther. Med. 16 (6), 4817–4823. 10.3892/etm.2018.6755 30542437 PMC6257662

[B22] Qin Y. He Y. H. Hou N. Zhang G. S. Cai Y. Zhang G. P. (2016). Sonic hedgehog improves ischemia-induced neovascularization by enhancing endothelial progenitor cell function in type 1 diabetes. Mol. Cell. Endocrinol. 423, 30–39. 10.1016/j.mce.2016.01.005 26773732

[B23] Sharifi-Rad M. Varoni E. M. Iriti M. Martorell M. Setzer W. N. Del Mar Contreras M. (2018). Carvacrol and human health: a comprehensive review. Phytother. Res. 32 (9), 1675–1687. 10.1002/ptr.6103 29744941

[B24] Shoorei H. Khaki A. Khaki A. A. Hemmati A. A. Moghimian M. Shokoohi M. (2019). The ameliorative effect of carvacrol on oxidative stress and germ cell apoptosis in testicular tissue of adult diabetic rats. Biomed. Pharmacother. 111, 568–578. 10.1016/j.biopha.2018.12.054 30597310

[B25] Siddall H. K. Warrell C. E. Yellon D. M. Mocanu M. M. (2008). Ischemia-reperfusion injury and cardioprotection: investigating PTEN, the phosphatase that negatively regulates PI3K, using a congenital model of PTEN haploinsufficiency. Basic Res. Cardiol. 103 (6), 560–568. 10.1007/s00395-008-0735-y 18604624

[B26] Szablewski L. (2017). Glucose transporters in healthy heart and in cardiac disease. Int. J. Cardiol. 230, 70–75. 10.1016/j.ijcard.2016.12.083 28034463

[B27] Xiao Q. Hou N. Wang Y. P. He L. S. He Y. H. Zhang G. P. (2012). Impaired sonic hedgehog pathway contributes to cardiac dysfunction in type 1 diabetic mice with myocardial infarction. Cardiovasc. Res. 95 (4), 507–516. 10.1093/cvr/cvs216 22745384

[B28] Yao H. Han X. Han X. (2014). The cardioprotection of the insulin-mediated PI3K/Akt/mTOR signaling pathway. Am. J. Cardiovasc. Drugs 14 (6), 433–442. 10.1007/s40256-014-0089-9 25160498

[B29] Yuan X. Xiao Y. C. Zhang G. P. Hou N. Wu X. Q. Chen W. L. (2016). Chloroquine improves left ventricle diastolic function in streptozotocin-induced diabetic mice. Drug Des. Devel. Ther. 10, 2729–2737. 10.2147/DDDT.S111253 PMC5012595 27621594

[B30] Zhao G. J. Hou N. Cai S. A. Liu X. W. Li A. Q. Cheng C. F. (2018). Contributions of Nrf2 to puerarin prevention of cardiac hypertrophy and its metabolic enzymes expression in rats. J. Pharmacol. Exp. Ther. 366 (3), 458–469. 10.1124/jpet.118.248369 29945930

