# Supplementary material for: Carvacrol Attenuates Diabetic Cardiomyopathy by Modulating the PI3K/AKT/GLUT4 Pathway in Diabetic Mice
Source: Front Pharmacol. 2019 Sep 12;10:998. doi: 10.3389/fphar.2019.00998 (PMC6751321; doi:10.3389/fphar.2019.00998)
Supplement: Supplementary file 1 [file DataSheet_1.pdf]

Supplements

Table S1. Antibody list

Antibody Name	Catalog Number	Manufactures	Application and dilution
p-PI3Kp85 (Tyr458)	4228	Cell signaling technology	WB (1 : 1000)
PI3Kp85	4257	Cell signaling technology	WB (1 : 1000)
GAPDH	AP0066	Bioworld technology	WB (1 : 10000)
p-PDK1 (Ser241)	3438	Cell signaling technology	WB (1 : 1000)
PDK1	BS1291	Bioworld technology	WB (1 : 1000)
p-PTEN (Ser380/Thr382/383)	9549	Cell signaling technology	WB (1 : 1000)
PTEN	BS1303	Bioworld technology	WB (1 : 1000)
p-AKT(Ser473)	4060	Cell signaling technology	WB (1 : 3000)
AKT	4691	Cell signaling technology	WB (1 : 3000)
p-AS160 (Thr642)	8881	Cell signaling technology	WB (1 : 1000)
AS160	2670	Cell signaling technology	WB (1 : 1000)
Sodium Potassium ATPase	ab76020	Abcam	WB (1 : 10000)
Glut4	BS3680	Bioworld technology	WB (1 : 1000)
Glut4	NBP1-49533	Novus biologicals	IF (1:100)

Supplementary figures

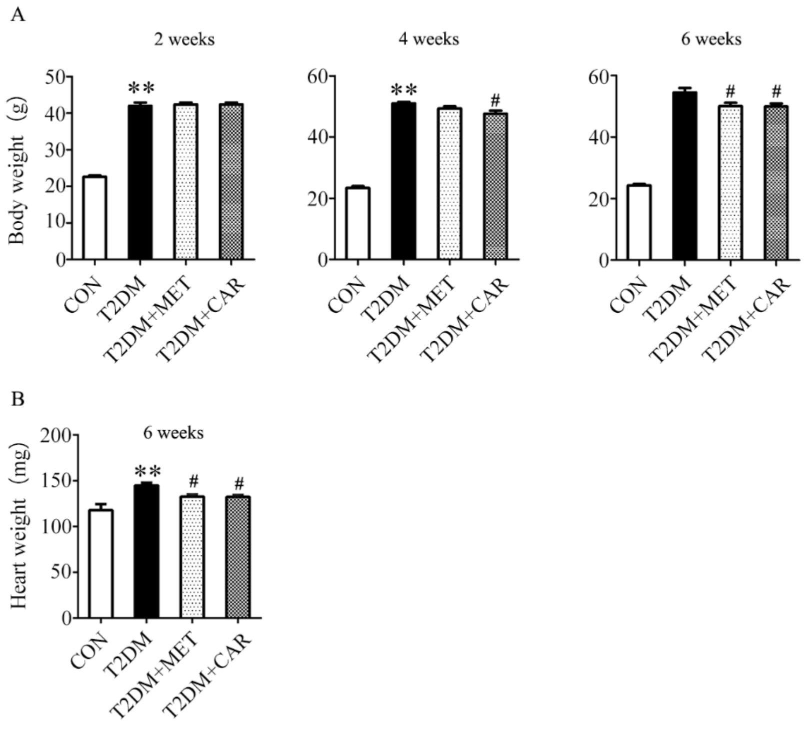

Figure S1. Effects of Carvacrol on body weight and heart weight in *db/db* mice. Con: control mice treated with vehicle (0.1% DMSO); T2DM: *db/db* mice treated with vehicle (0.1% DMSO); T2DM+MET: *db/db* mice treated with 100 mg/kg/day metformin; T2DM+CAR: *db/db* mice treated with 20 mg/kg/day Carvacrol. Data was presented as mean ± SEM. **P* < 0.05, ***P* < 0.01 VS. Con group; #*P* < 0.05, ##*P* < 0.01 VS. T2DM group.

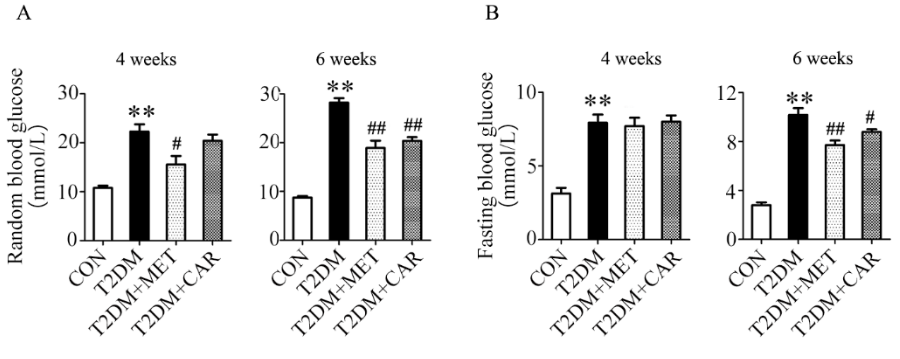

Figure S2. Effects of Carvacrol on random blood glucose, and fasting blood glucose in in *db/db* mice. Con: control mice treated with vehicle (0.1% DMSO); T2DM: *db/db* mice treated with vehicle (0.1% DMSO); T2DM+MET: *db/db* mice treated with 100 mg/kg/day metformin; T2DM+CAR: *db/db* mice treated with 20 mg/kg/day Carvacrol. Data was presented as mean ± SEM. **P* < 0.05, ***P* < 0.01 VS. Con

group; #$P < 0.05$, ##$P < 0.01$ VS. T2DM group.
